# A Population-Based Scoring System to Assess the Impact of Individual Risk Factors on Vascular Health

**DOI:** 10.14336/AD.2023.0823

**Published:** 2024-05-07

**Authors:** Vanessa Gagliano, David Gehrig, Rosaria Del Giorno, Jvan Gianini, Luca Gabutti

**Affiliations:** ^1^Department of Internal Medicine, Clinical Research Unit, Regional Hospital of Bellinzona and Valli, Ente Ospedaliero Cantonale, Bellinzona, Switzerland.; ^2^Faculty of Biomedicine, Università della Svizzera Italiana, Lugano, Switzerland.; ^3^Angiology service, University Hospital of Lausanne, Lausanne, Switzerland.

**Keywords:** Pulse wave velocity, nomogram, cardiovascular risk factors, arterial stiffness, vascular aging

## Abstract

Arterial stiffness is an indicator of vascular health, influenced by both pathological conditions and physiological determinants, noticeably age. Augmentation index (AI) and pulse wave velocity (PWV) are used among others to assess arterial stiffness. Several risk factors may contribute to pathologically increase arterial stiffness and produce early vascular aging. Our study aims to assess the impact of individual risk factors on vascular health, evaluating the distribution of PWV and AI values in a cohort of adult people without modifiable cardiovascular risk factors while analyzing their role in accelerating vascular ageing. We performed a secondary analysis of a Swiss population-based research project, which took place in 2017 and 2018. Of the 1202 participants originally enrolled, 1097 were included in the final sample. The population was divided into without (n=388) and with risk factors (n=709), based on the presence of the following: smoking, diabetes, previous cardiovascular disease (CVD), chronic kidney disease stage 3 or more, LDL cholesterol ≥ 4.11 or treatment with hypolipidemic drugs, hypertension or treatment with antihypertensive drugs, and metabolic syndrome. Tonometric and oscillometric devices were employed to assess PWV, and the 75th percentiles of PWV and AI in the population without risk factors were calculated to identify cut-offs for the logistic regression analysis. We developed nomograms by assigning a numerical score to each independent prognostic factor; the total score estimating the probability of PWVs and AIs being over the defined cut-offs. Patients with hypertension, diabetes, and obesity showed higher PWV values (p < 0.001). In the univariate logistic regression, factors predictive for higher PWV values were diabetes, CVDs, hypercholesterolemia, and hypertension, while CVDs, antihyperlipidemic treatment, hypertension, and increased BMI were predictive in the multivariate logistic regression. Smoking did not significantly influence arterial stiffness parameters. The present study provides reference values for PWV and AI in subjects without modifiable cardiovascular risk factors and, through nomograms, a risk score stratification to assess the impact of individual risk factors on vascular health.

## Introduction

Cardiovascular diseases (CVDs) are the leading cause of mortality and disability worldwide [1]. A measurable index of vascular health is arterial stiffness, which represents an important predictor of cardiovascular events and cognitive decline even in asymptomatic individuals with no overt CVD [2-6], and could play an important role in disease prevention and risk stratification.

When arteries become less elastic and distensible, their hemodynamic properties change, resulting in reduced compliance and buffering capacity to pulsatile cardiac ejection [7]. Notably, arterial stiffening is influenced by physiological determinants such as age, sex, and blood pressure [8-10], and by pathological conditions, including hypertension, renal failure, diabetes, obesity, dyslipidemia and the metabolic syndrome [11-17], while the effects of smoking still remain controversial [18, 19]. Aging, on the other hand, is recognized to have the most impact overall [20-22], inducing functional and structural alterations in the vascular walls of both central and peripheral arteries, causing them to be thicker and greater in conduit diameter as time goes on. However, since the repeated exposure to potentially harmful stimuli across the lifespan could further hamper vascular health and produce inter-individual differences, it is noteworthy that the chronological age of a person does not always reflect the actual biological age of the arteries and that favorable vascular aging parameters can also occur in the presence of significant CV risk factors [17, 23]. This concept is referred to as early vascular ageing (EVA), and healthy vascular ageing (HVA), with PWV values in the lowest and highest 10% of the population distribution respectively [17].

The two most reliable parameters used to assess arterial stiffness clinically are pulse wave velocity (PWV), representing the rate at which the blood pressure pulse propagates down the circulatory system, and augmentation index (AI), describing the effect of systolic wave reflection on the central aorta. To date, the gold standard for non-invasive estimation of arterial stiffness is the tonometric measurement of carotid-femoral PWV (cf-PWV) [24], which strongly correlates with the incidence of CVDs independently from traditional risk factors, but it is time-consuming and requires sophisticated equipment and highly trained operators. Therefore, oscillometric-based devices, capturing brachial blood pressure and waveforms, and the cardio-ankle vascular index (CAVI), have been proposed as valid and operator-independent alternatives for arterial stiffness evaluation in the daily clinical practice [16, 25].

PWV and AI cannot be used interchangeably [26, 27], and data about their population specific reference values are still incomplete due to a limited number of large studies [28] and the lack of standardization of the measurement methods [29, 30]. The 2007 ESH/ESC hypertension guidelines first proposed a fixed age independent pathological threshold value of 12 m/s for PWV; however, it did not take into account the multiple factors influencing it [31]. In 2010, an extensive European study gathered data from 16’867 subjects and was able to establish reference values for PWV, based on age and blood pressure categories, in a healthy population [32]. Later on, similar investigations were conducted in Spain [33], China [34, 35], USA [21, 36], South America [37] and in transcontinental collaborations [17].

Our study aims to establish age specific reference values of PWV and AI in a cohort of adults without modifiable CV risk factors, and to quantitatively evaluate the impact of individual cardiovascular risk factors on both parameters.

## MATERIALS AND METHODS

### Study design

This study was based on a cross-sectional analysis of a population-focused research project (Ticino Epidemiological Stiffness Study; TEST-study), which took place in Southern Switzerland between the years 2017 and 2018.

### Ethical approval

The study has received approval by the Ethics Committee with the number 2016-01718 [38]. The study was performed in accordance with the Helsinki Declaration of 1964, and its later amendments. All subjects provided informed consent to participate in the study.

### Sample

**Figure 1.**
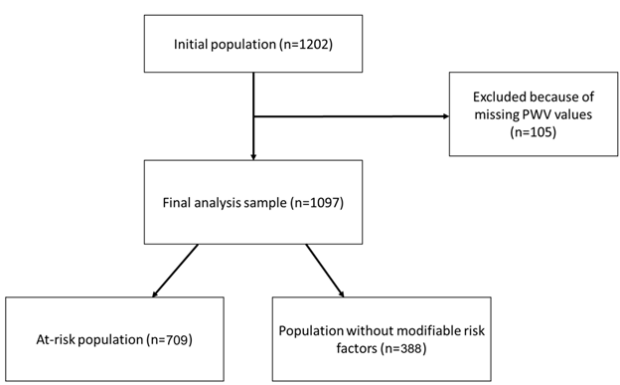
Flowchart showing the selection procedure of the participants.

The original study counted a total of 1202 participants, but only those who had complete data available in regard to the assayed variables were finally included in the present analysis (n=1097) (Fig. 1).

The population was divided into the population without modifiable risk factors (called “normal”) and the at-risk population, i.e., patients with one or more of the following risk factors: smoking, diabetes, previous CVD, Chronic Kidney Disease (CKD) stage 3 or more, LDL ≥ 4.1 mmol/L, hypertension, use of hypolipidemic and antihypertensive drugs, and metabolic syndrome.

The CKD-EPI Creatinine Equation 2021 was used to estimate GFR and to classify subjects in CKD stages 1-5 [39].

According to the NCEP ATP III definition, metabolic syndrome was diagnosed if three or more of the five key criteria were met: waist circumference >101.6 cm (men) or 88.9 cm (women), blood pressure >130/85 mmHg, fasting triglyceride level >1.69 mmol/L, fasting high-density lipoprotein (HDL) cholesterol level <1.03 mmol/L (men) or 1.29 mmol/L (women) and fasting blood glucose over 5.5 mmol/L [40]. In case of the unavailability of a fasting blood glucose, a HbA1c value of 5.7% was used as the cut-off [41] .

The cut-off for LDL was chosen according to the 2018 American Guideline on the Management of blood cholesterol, where primary hypercholesterolemia (LDL 4.1-4.8 mmol/l; non-HDL 4.9-5.6 mmol/l) listed among the risk-enhancing factors for initiation of statin therapy, in adults 40-75 years of age without diabetes mellitus and with intermediate risk for CV events according to the Framingham Risk Score [42]. Familiarity for cardiovascular diseases was not considered in the selection criteria because it is an unmodifiable risk factor.

Our aim was to evaluate the age specific distribution of PWV and AI values in a population without modifiable risk factors and to evaluate the quantitative impact of individual risk factors on vascular stiffness.

### Instruments

Two different methods and devices were used in order to obtain PWV values: oscillometric (Mobil-O-Graph, Industrielle Entwicklung Medizintechnik und Vertriebsgesellschaft, Germany; brachial pulse wave analysis; br-PWV) and tonometric (SphygmoCor, Atcor, CardieX Limited, Australia; carotid-femoral pulse wave determination; cf-PWV) [25]. A good agreement between oscillometric and tonometric PWV values in the same study population was documented and published by our group in 2020 [25]. AI values were acquired via the SphygmoCor device alone.

### Statistical Analyses

Data were summarized as mean and standard deviation for quantitative variables and as frequencies and percentages for qualitative variables. For cf-PWV, br-PWV, and AI, percentiles for the overall sample, at-risk and “normal” population, gender, and age ranges were also determined. Comparisons between normal- and at-risk-population were performed through the Student t and chi-square tests.

The 75th percentiles of the normal population of cf-PWV, br-PWV, and AI were used to identify cut-offs to create three dummy variables, subsequently used as dependent variables in univariate and multivariate logistic regression models. Nomograms were drawn after logistic regressions, including the following risk factors BMI, gender, CVD familiarity, previous CVD, hypertension, diabetes, and hypolipidemic drugs, and were developed by assigning a numerical score to each independent prognostic factor, using the total score to assess the probability of having PWV and AI values higher than the defined cut-offs. Statistical analyses were performed through STATA17 (StataCorp. College Station, TX, USA). Statistical significance was set at 5% (p < 0.05).

## RESULTS

One thousand and ninety-seven subjects were included in the study. According to previous criteria, 709 were classified as “with modifiable risk factors”, and the remaining 388 were instead classified as “without modifiable risk factors”. The mean age of the sample was 54.4 ± 13.7 years and resulted higher in the “at-risk” population than in the “normal” one (p < 0.001). About 56.3% of the sample was composed of females, with a statistically significant difference between the two populations (p ≤ 0.001) given the high concentration of females in the normal group (70% vs. 48.8%). The at-risk population was taller (169.1 ± 9.5 cm), with higher body weight (74.3 ± 16.5 kg), than the “normal” subjects (p < 0.05). This difference, in turn, had repercussions on BMI, which was higher in subjects at-risk (p < 0.001). Approximately half of the subjects performed physical activity twice a week, while 23.7% stated not practicing physical activity; at-risk subjects showed less physical activity than normal (p < 0.01). Mean SBP and DBP values were significantly higher in the at-risk population (SBP: 125.4 ± 12.6 mmHg, DBP: 79.1 ± 9.5 mmHg) than in the normal one (SBP: 115.4 ± 7.9 mmHg; DBP: 72.5 ± 6.2 mmHg) (p < 0.001); higher total cholesterol (5.6 ± 1.6 mmol/L) and triglycerides (1.3 ± 1.6 mg/dL) were also observed in at-risk patients. “At-risk” subjects also showed significantly higher LDL and lower HDL cholesterol values (p < 0.001). Cardiovascular disease familiarity was more common in the at-risk population (21.5%) than in the “normal” one (14.4%) (p < 0.001). Br-PWV, and cf-PWV were higher in at-risk subjects (br-PWV: 7.7 ± 1.9; cf-PWV: 7.8 ± 1.8) than in the normal population (br-PWV: 6.5 ± 1.3, cf-PWV: 6.5 ± 1.2) (p < 0.001). Complete subject characteristics are shown in Table 1. Percentile distribution for cf-PWV, br-PWV, and AI are also shown in Table 2 and Figure 1.

**Table 1:** Subjects’ characteristics and differences between the populations with and without modifiable risk factors.

	All Sample	At-risk population	Population without modifiable risk factors	p
**Number of Subjects**	1097	709	388	
**Age (years), mean (SD)**	54.4 (13.7)	57.5 (13.7)	48.7 (11.8)	0.000*
**Age range, n (%)**				
***20-29***	36 (3.3)	17 (2.4)	19 (4.9)	0.000^§^
***30-39***	125 (11.4)	51 (7.2)	74 (19.0)	
***40-49***	224 (20.4)	122 (17.1)	102 (26.4)	
***50-59***	349 (31.8)	209 (29.4)	140 (36.2)	
***60-69***	197 (18.0)	166 (23.4)	31 (8.0)	
***70-79***	119 (10.9)	99 (14.0)	20 (5.1)	
***≥ 80***	47 (4.3)	45 (6.4)	2 (0.5)	
**Gender, %**				
***Female***	618 (56.3)	346 (48.8)	272 (70.0)	0.000*
***Male***	479 (43.7)	363 (51.2)	116 (30.0)	
**Height (cm), mean (SD)**	168.7 (9.4)	169.1 (9.6)	167.9 (9.1)	0.019*
**Weight (kg), mean (SD)**	71.6 (15.9)	74.3 (16.5)	66.5 (13.1)	0.000*
**Waist circumference (cm), mean (SD)**	88.7 (14.6)	91.5 (15.5)	83.7 (11.4)	0.668*
**Hips circumference (cm), mean (SD)**	97.5 (12.5)	98.75 (13.4)	95.2 (10.2)	0.857*
**BMI, mean (SD)**	25.0 (4.4)	25.9 (4.6)	23.5 (3.5)	0.000*
**BMI ≥ 30, n (%)**	134 (12.2)	113 (15.8)	21 (5.64)	NA
**Alcohol consumption, n (%) (n = 1065)**	860 (80.8)	562 (82.1)	298 (78.3)	NA
**Number of daily coffees, n (%)** *				
***No coffee***	109 (10.1)	62 (8.9)	47 (12.2)	0.054^§^
***1-3***	766 (71.0)	486 (70.0)	280 (72.7)	
***4-6***	175 (16.2)	126 (18.2)	49 (12.7)	
***> 6***	29 (2.7)	20 (2.9)	9 (2.3)	
**Daily Walking (minutes), mean (SD) (n = 1022)**	65.1 (85.7)	65.6 (89.0)	64.3 (79.6)	0.183*
**Physical activity, n (%) (n = 1046)**				
***Once a week***	165 (15.8)	103 (15.3)	62 (16.6)	0.001^§^
***Twice a week***	501 (47.9)	293 (43.8)	208 (55.2)	
***Once a month***	64 (6.1)	45 (6.8)	19 (5.0)	
***At least once a month***	68 (6.5)	43 (6.5)	25 (6.6)	
***Never***	248 (23.7)	185 (27.7)	63 (16.6)	
**SBP (mmHg), mean (sd)**	121.9 (12.1)	125.4 (12.6)	115.4 (7.9)	0.000*
**DBP (mmHg), mean (sd)**	76.8 (9.0)	79.1 (9.5)	72.5 (6.2)	0.000*
**Hypertension, n (%)**	269 (24.5)	269 (38.1)	0 (0)	
**Antihypertensive drugs, n (%)**	176 (16.0)	176 (24.9)	0 (0)	
**Total cholesterol (mmol/L), mean (sd)**	5.4 (1.4)	5.6 (1.6)	4.9 (0.8)	0.000*
**LDL cholesterol (mmol/L), mean (sd)**	3.6 (1.0)	3.9 (1.1)	3.1(0.7)	0.000*
**HDL cholesterol (mmol/L), mean (sd)**	1.6 (0.4)	1.5 (0.4)	1.7 (0.4)	0.000*
**Triglycerides (mg/dL), mean (sd)**	1.2 (1.4)	1.3 (1.6)	0.8 (0.6)	0.000*
**Hypolipidemic drugs, n (%)**	166 (15.1)	166 (23.5)	0 (0)	
**Diabetes, n (%)**	20 (1.8)	20 (2.8)	0 (0)	
**Antidiabetic drugs, n (%)**	20 (1.8)	20 (2.8)	0 (0)	
**CVD familiarity, n (%)**	208 (19.0)	154 (21.5)	54 (14.4)	0.000
**Previous CVD, n (%)**	38 (3.5)	38 (5.4)	0 (0)	
**Metabolic syndrome, n (%)**	43 (3.9)	43 (6.1)	0 (0)	
**CKD Stage 3 or more**	35 (1.7)	35 (4.9)	0 (0)	
**Br-PWV) (m/s), mean (SD)**	7.6 (1.8)	7.7 (1.9)	6.5 (1.3)	0.000*
**AI, mean (SD)**	22.8 (13.8)	23.3 (14.0)	21.7 (13.4)	0.047*
**Cf-PWV, mean (SD)**	7.3 (1.7)	7.8 (1.8)	6.5 (1.2)	0.000*

* Student t-test,

§ Chi-square test

Subjects with hypertension reported significantly higher br-PWV (8.34 ± 1.70), cf-PWV (8.29 ± 1.66), and augmentation index (24.62 ± 13.39) values (p < 0.01), while diabetics and obese subjects showed significant higher br- and cf-PWV values (p < 0.001) (Table 3).

From univariate logistic regression (Table 4), the following variables results were predictive for br-PWV and cf-PVW higher than 6.9 and 7.1 m/s: diabetes (OR: 1.9, 95%CI: 1.14 - 2.71; OR 1.98, 95%CI: 1.22 - 2.73), previous cardiovascular disease (OR: 2.73, 95%CI: 2.18 - 3.29; OR: 2.69, 95%CI: 2.15 - 3.23), use of hypolipidemic drugs (OR: 1.99, 95%CI: 1.72 - 2,26; OR: 1.80, 95%CI: 1.54 - 2.07), hypertension (OR: 1.24, 95%CI: 1.01 - 1.48; OR: 1.26, 95%CI: 1.04 - 1.49), use of hypertension drugs (OR: 2.43, 95%CI: 2.18 - 2.68; OR: 2.23, 95%CI: 1.98 - 2.47), increase in total cholesterol (OR: 0.13, 95%CI: 0.05 - 0.20; OR: 0.11, 95%CI: 0.04 - 0.18),and LDL cholesterol (OR: 0.28, 95%CI: 0.18 - 0.38, OR: 0.26, 95%CI: 0.17 - 0.36); while hypertension (OR: 2.54, 95%CI: 0.64 - 4.43) and HDL cholesterol (OR: 4.13, 95%CI: 2.24 - 6.02) resulted predictive for augmentation indexes higher than 32.

**Table 2:** Percentiles distribution for cf-PWV, br-PWV, and augmentation index.

		5^th^	10^th^	25^th^	50^th^	75^th^	90^th^	95^th^
**br-PWV**	**Overall Sample**	5.1	5.3	6.1	7.1	8.3	10.0	11.0
	**At-risk Population**	5.2	5.7	6.5	7.6	9.0	10.6	11.4
	**“Normal” Population**	5.0	5.2	5.6	6.3	7.1	8.2	9.4
	**Gender**							
	Females	5.0	5.2	5.9	7.0	8.2	9.8	10.8
	Males	5.3	5.6	6.3	7.2	8.5	10.2	11.1
	**Age Ranges**							
	20-29 years	4.5	4.5	4.6	4.9	5.2	5.5	5.8
	30-39 years	4.9	4.9	5.1	5.3	5.5	5.9	6.0
	40-49 years	5.5	5.6	5.8	6.1	6.4	6.7	6.9
	50-59 years	6.2	6.4	6.8	7.1	7.5	7.8	8.0
	60-69 years	7.4	7.7	8.0	8.4	8.8	9.4	9.6
	70-79 years	9.0	9.3	9.6	10.0	10.5	11.0	11.4
	≥ 80 years	11.1	11.1	11.3	11.9	12.4	12.8	13.2
**AI**	**Overall Sample**	0.0	2.0	11.0	25.0	34.0	40.0	42.0
	**At-risk Population**	-1.0	2.0	12.0	25.0	35.0	40.0	43.0
	**“Normal” Population**	0.0	3.0	10.0	24.0	32.0	38.0	41.0
	**Gender**							
	Females	3.0	7.0	15.0	29.0	36.0	41.0	44.0
	Males	-3.0	0.0	6.0	20.0	29.0	36.0	39.0
	**Age Ranges**							
	20-29 years	2.0	3.0	8.5	22.5	28.5	33.0	35.0
	30-39 years	0.0	1.0	10.0	23.0	31.0	39.0	41.0
	40-49 years	-2.0	1.0	11.5	26.5	34.0	40.0	42.0
	50-59 years	-1.0	2.0	10.0	22.0	33.0	38.0	41.0
	60-69 years	-1.0	3.0	13.0	27.0	37.0	41.0	44.0
	70-79 years	1.0	4.0	15.0	28.0	37.0	41.0	45.0
	≥ 80 years	2.0	7.0	15.0	32.0	37.0	40.0	42.0
**cf-PWV**	**Overall Sample**	5.1	5.4	6.1	6.9	8.3	9.8	10.9
	**At-risk Population**	5.3	5.7	6.6	7.5	8.7	10.4	11.5
	**“Normal” Population**	4.8	5.1	5.7	6.4	6.9	8.0	8.9
	**Gender**							
	Females	5.0	5.3	6.0	6.8	8.0	9.6	10.7
	Males	5.3	5.7	6.4	7.3	8.5	9.9	11.2
	**Age Ranges**							
	20-29 years	4.1	4.4	4.6	5.1	5.4	5.8	6.3
	30-39 years	4.7	4.8	5.0	5.4	5.9	6.3	6.7
	40-49 years	5.2	5.4	5.8	6.2	6.6	7.0	7.3
	50-59 years	6.0	6.2	6.6	6.9	7.5	8.1	8.4
	60-69 years	6.8	7.0	7.6	8.1	8.7	9.3	9.8
	70-79 years	8.4	8.6	9.1	9.6	10.5	11.4	11.8
	≥ 80 years	10.6	10.6	11.0	11.6	12.3	12.9	13.2

From multivariate logistic regressions, PWV resulted statistically influenced by several factors: use of hypolipemic drugs (br-PWV: OR: 7.34, 95%CI: 4.40-12.24; cf-PWV: OR: 7.65, 95%CI: 4.38 -13.36; p < 0.001), hypertension (br-PWV: OR: 4.16, 95%CI: 2.95-5.87; cf-PWV: OR: 4.79; 95%CI: 3.32 - 6.93; p < 0.001), cardiovascular diseases (br-PWV: OR: 11.62, 95%CI: 1.15-89.15; cf-PWV: OR: 8.60, 95%CI: 1.11 - 66.45; p< 0.05), and increase in BMI (br-PWV: OR: 1.07, 95%CI: 1.03-1.10; cf-PWV: OR: 1.08; 95%CI: 1.05-1.12; p < 0.001). For the augmentation index, males showed a reduced risk of high AI values compared to females (OR: 0.24, 95%CI: 0.17-0.33, p < 0.001), while hypertensive subjects reported a higher risk of AI values above 32 (OR: 1.93, 95%CI: 1.39-2.69, p < 0.001).

**Figure 2.**
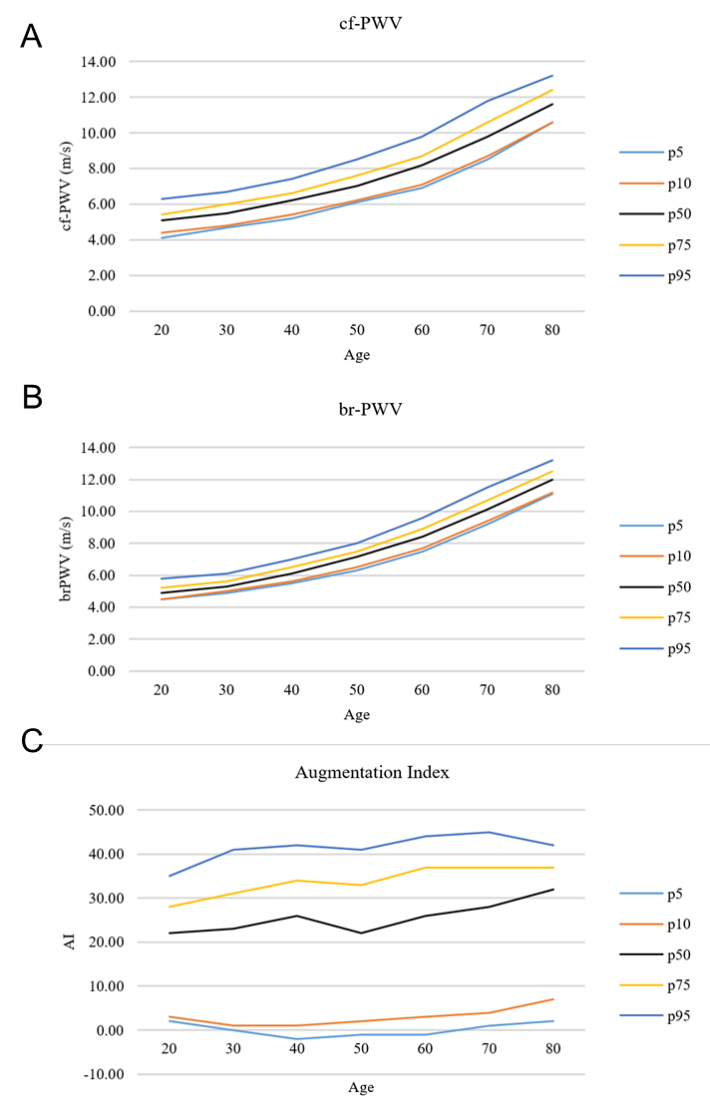
Graphical distribution of cf-PWV (A), br-PWV (B), and AI (C) according to age.

Nomograms were developed by assigning a numerical score to each independent prognostic factor. A higher total score was associated with a higher probability of having PWV or AI, higher than the defined cut-offs (Fig. 2).

**Table 3:** Differences in parameters between individuals with and without risk factors.

	Hypertension			Diabetes			Obesity (BMI ≥ 30)		
	Yes	No	p	Yes	No	p	Yes	No	p
**br-PWV, mean (sd)**	8.34 (1.70)	7.10 (1.71)	0.000	9.30 (1.70)	7.37 (1.77)	0.000	8.17 (1.78)	7.30 (1.77)	0.000
**AI, mean (sd)**	24.62 (13.39)	22.09 (13.89)	0.009	28.10 (12.35)	22.61 (13.82)	0.078	23.54 (13.77)	22.59 (13.81)	0.455
**cf-PWV, mean (sd)**	8.29 (1.66)	7.03 (1.63)	0.000	9.28 (1.82)	7.30 (1.71)	0.000	8.06 (1.77)	7.24 (1.70)	0.000

## DISCUSSION

The present population-based study focuses on the evaluation of age specific PWV and augmentation index parameters in a sub-population of subjects without modifiable risk factors and on their individual role in accelerating vascular aging. Considering that there are many potential determinants of arterial stiffening, both physiological and pathological, we deemed it of interest to dispose of a nomogram, which links the exposure to potentially harmful and correctable risk factors to the probability of having higher than expected, age specific PWV and AI values.

**Table 4:** Univariate Regressions in the Total Population.

	br-PWV (cut-off: 6.9)	AI (cut-off: 32)	cf-PWV (cut-off: 7.1)
**Smoking**	0.01 (0.11) (-0.20; 0.23)	-0.52 (0.84) (-2.16; 1.12)	-0.02 (0.11) (-0.23; 0.19)
**Diabetes**	1.9*** (0.40) (1.14 2.71)	5.49 (3.11) (-0.62; 11.60)	1.98*** (0.39) (1.22; 2.73)
**CVD**	2.73*** (0.28) (2.18; 3.29)	0.06 (2.28) (-4.42; 4.53)	2.69*** (0.27) (2.15; 3.23)
**CVD Familiarity**	0.27 (0.14) (-0.01; 0.54)	-1.64 (1.06) (-3.73; 0.45)	0.22 (0.13) (-0.04; 0.48)
**Hypolipidemic drugs**	1.99*** (0.14) (1.72; 2.26)	1.75 (1.16) (-0.53; 4.03)	1.80*** (0.14) (1.54; 2.07)
**Hypertension**	1.24*** (0.12) (1.01; 1.48)	2.54*** (0.97) (0.64; 4.43)	1.26*** (0.12) (1.04; 1.49)
**Hypertension drugs**	2.43*** (0.13) (2.18; 2.68)	0.50 (1.14) (-1.73; 2.73)	2.23*** (0.13) (1.98; 2.47)
**Total cholesterol (mmol/L)**	0.13*** (0.04) (0.05; 0.20)	0.28 (0.30) (-0.30; 0.86)	0.11*** (0.04) (0.04; 0.18)
**HDL cholesterol (mmol/L)**	-0.19 (0.13) (-0.44; 0.05)	4.13*** (0.96) (2.24; 6.02)	-0.31** (0.12) (-0.54; -0.07)
**LDL cholesterol (mmol/dL)**	0.28*** (0.05) (0.18; 0.38)	0.38 (0.40) (-0.41; 1.17)	0.26*** (0.05) (0.17; 0.36)

**Figure 3.**
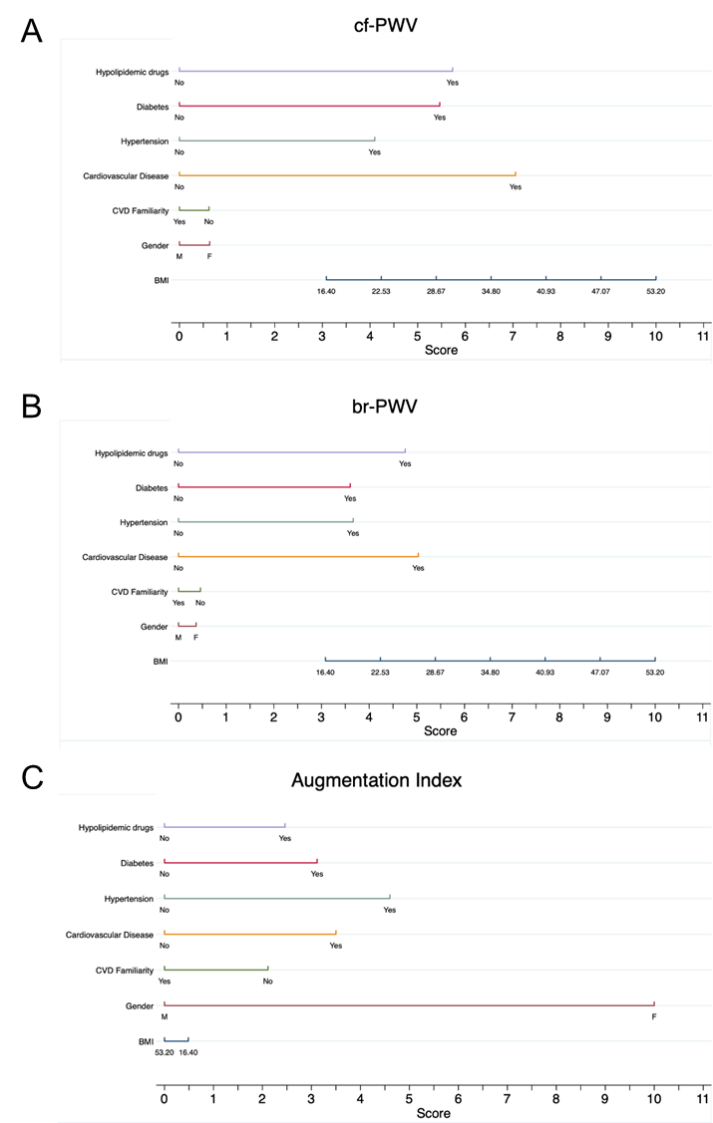
**Nomograms assign numerical scores to each independent prognostic factor**. A higher total score is associated with a higher probability (prob) of having cf-PWVs (A), br-PWVs (B) or AIs (C) above the 75^th^ percentile.

For the purpose of our study, we took into account the main cardiovascular risk factors, and we investigated their relationship with the oscillometric brachial and the tonometric carotid-femoral PWV (br-PWV and cf-PWV respectively) and with AI. In addition, since data in the literature about reference values of PWV and AI in subjects without modifiable risk factors is limited, we examined their distribution in a cohort of healthy adults, according to age and sex.

Our study sample was divided into two groups for the statistical analysis: the population without modifiable risk factors (n=388) and the at-risk population (n=709), i.e., patients with one or more of the following risk factors: smoking, diabetes, previous CVD, CKD stage 3 or more, LDL ≥ 4.1 mmol/L, hypertension, use of hypolipidemic and antihypertensive drugs, and metabolic syndrome.

Results showed that br-PWV, and cf-PWV were higher in at-risk subjects (br-PWV: 7.7 ± 1.9; cf-PWV: 7.8 ± 1.8) than in the population without risk factors (br-PWV: 6.5 ± 1.2, cf-PWV: 6.5 ± 1.2) (p < 0.001). Moreover, from univariate logistic regression, diabetes, previous cardiovascular disease, use of hypolipidemic drugs, hypertension, use of hypertension drugs, increase in total cholesterol and LDL cholesterol were predictive for br-PWV and cf-PVW, higher than 6.9 and 7.1 m/s. Instead, hypertension and HDL cholesterol were predictive for AI higher than 32. Not surprisingly, considering the contradictory data in the literature, we did not demonstrate higher PWV values in smokers; a finding, however, that must be regarded with caution given the size of the at-risk population studied.

Finally, we developed nomograms assigning a numerical score to each independent prognostic factor, so that a higher total score is associated with a higher probability of having PWVs or AIs above the defined cut-offs. Nomograms are mainly intended as tools to be employed by general practitioners for primary assessment of patients and to aid in the decision for more aggressive treatment whenever needed. Considering the fact that in most cases, automated oscillometric devices are used for PWV determination in clinical practice, we provide both oscillometric and tonometric data analysis.

Among the limitations of our study, there is the choice of the population (a Swiss Caucasian) mainly representative of central Europe, and the narrow sample size especially of the subgroup without modifiable risk factors and for individual risk factors.

Nonetheless, we offer an in-depth analysis based on both tonometric and oscillometric PWV measurements in a population where the reproducibility of the oscillometric method, using the tonometric as the gold standard, was already assessed by our group [25].

In conclusion, the present study, based on a sample of the Swiss population without modifiable risk factors, provides reference PWV values obtained tonometrically by carotid-femoral determination (cf-PWV) and oscillometrically by brachial pulse wave analysis (br-PWV), and AI values, according to internally and externally validated methodologies.

These results help in the risk stratification of individual subjects in relation to their age, sex, and the presence of cardiovascular risk factors. Nomograms and percentile reference ranges can be used as tools for the identification of people who might benefit from more comprehensive follow-up. Nonetheless, larger population-based studies focused on the same subject would be helpful in order to shed further light on this relevant public health topic.
